# Enhancing Web-Based Mindfulness Training for Mental Health Promotion With the Health Action Process Approach: Randomized Controlled Trial

**DOI:** 10.2196/jmir.3746

**Published:** 2015-01-19

**Authors:** Winnie WS Mak, Amy TY Chan, Eliza YL Cheung, Cherry LY Lin, Karin CS Ngai

**Affiliations:** ^1^Diversity & Well-Being LaboratoryDepartment of PsychologyThe Chinese University of Hong KongShatin, NT, Hong KongChina (Hong Kong)

**Keywords:** Internet-based intervention, online intervention, mindfulness, Health Action Process Approach (HAPA), mental health promotion

## Abstract

**Background:**

With increasing evidence demonstrating the effectiveness of Web-based interventions and mindfulness-based training in improving health, delivering mindfulness training online is an attractive proposition.

**Objective:**

The aim of this study was to evaluate the efficacy of two Internet-based interventions (basic mindfulness and Health Action Process Approach enhanced mindfulness) with waitlist control. Health Action Process Approach (HAPA) principles were used to enhance participants’ efficacy and planning.

**Methods:**

Participants were recruited online and offline among local universities; 321 university students and staff were randomly assigned to three conditions. The basic and HAPA-enhanced groups completed the 8-week fully automated mindfulness training online. All participants (including control) were asked to complete an online questionnaire pre-program, post-program, and at 3-month follow-up.

**Results:**

Significant group by time interaction effect was found. The HAPA-enhanced group showed significantly higher levels of mindfulness from pre-intervention to post-intervention, and such improvement was sustained at follow-up. Both the basic and HAPA-enhanced mindfulness groups showed better mental well-being from pre-intervention to post-intervention, and improvement was sustained at 3-month follow-up.

**Conclusions:**

Online mindfulness training can improve mental health. An online platform is a viable medium to implement and disseminate evidence-based interventions and is a highly scalable approach to reach the general public.

**Trial Registration:**

Chinese Clinical Trial Registry (ChiCTR): ChiCTR-TRC-12002954; http://www.chictr.org/en/proj/show.aspx?proj=3904 (Archived by WebCite at http://www.webcitation.org/6VCdG09pA).

## Introduction

### Background

According to the World Health Organization [1], mental health is essential to contributing to the overall well-being of every individual. However, approximately 450 million people worldwide suffer from mental health problems, and they represent 15% of the total disease burden as assessed by disability-adjusted life years (DALY) [1-3]. Based on the Global Burden of Diseases, Injuries, and Risk Factors Study 2010 [4], depressive and anxiety disorders account for 55.1% of DALYs caused by mental and substance use disorders [5]. Given the tremendous burden on affected individuals, family, and society, it is critical for mental health promotion and prevention of mental illness to be incorporated into public health initiatives [6].

### Benefits of Internet-Based Interventions for Public Health Promotion

Considering that, in both primary and secondary care settings, there are tremendous service gaps between supply and demand for psychological services and the costs of treatment are large, Internet-based intervention is a viable option to reduce stress—which is a significant risk factor for poor mental health—and to promote mental well-being for adults in the community [1]. It provides an effective, low-cost, convenient, and anonymous alternative and makes health promotion more accessible to those who would otherwise not seek help due to cost, inconvenience, stigma, and other barriers to help-seeking [7,8]. In Hong Kong, the Internet is a convenient and accessible portal for providing health promotion and universal preventive measures, as 77.9% of households have Internet access and 72.8% of individuals over 10 years old have used the Internet in the past year [9].

Internet-based interventions have been shown to be effective in the prevention of depression and anxiety for both research trial participants and public registrants [10-16]. The effectiveness of Internet-based mental health promotion was also demonstrated in community samples in Western countries [10,12-14,16]; however, no study to date has evaluated Internet-based mental health promotion programs in Asia.

### Mindfulness as Means of Promoting Mental Health

In addition to the prevention of mental illness, mental health promotion involves tackling risk factors and promoting positive aspects of individuals’ lives and their overall quality of life [1]. In recent decades, mindfulness has gained attention in the domain of mental health promotion and psychological intervention. Many well-researched therapeutic approaches have incorporated mindfulness into their intervention, including acceptance and commitment therapy, dialectical behavior therapy, mindfulness-based cognitive therapy (MBCT), and mindfulness-based stress reduction (MBSR). Although these approaches may vary in terms of therapeutic techniques and emphasis, the core philosophy on present moment awareness with compassion remains the same. Traditionally, mindfulness is a systematic training to develop sustainable attention and awareness with the aim to glean insight from direct experiences [17]. It is part of the comprehensive Buddhist teachings at attaining genuine and sustainable well-being.

Mindfulness is an approach that focuses on the cultivation of conscious, non-judgmental awareness in the unfolding of events in the present moment [18]. It emphasizes the transience of all experiences. It involves self-regulation of attention and orientation towards the present moment with curiosity, openness, and acceptance [19]. Developed in the behavioral medicine setting for people with stress-related disorders, MBSR was the first manualized mindfulness-based training program. It was later adapted into MBCT [20]. During the 8-week program, mindfulness is cultivated through exercises such as sitting meditation, body scan, and stretching exercises [18]. In addition to formal practices, participants are encouraged to practice mindfulness during daily activities (eg, walking or eating).

Empirically, mindfulness-based approaches have demonstrated effectiveness in treating depression, anxiety disorders, and a host of other physical and mental health conditions in both non-clinical and clinical populations [21-25]. The approach also reduced psychological and physical symptoms among community adults [26-28]. A study conducted among adults in the wider community showed that, compared with cognitive-behavioral stress reduction, MBSR demonstrated larger effects across indices including energy, pain, well-being, and perceived stress [27]. In a randomized clinical trial comparing mindfulness with relaxation training among community adults, although both programs showed similar effects in reducing distress, mindfulness showed greater effects in enhancing positive states of mind [29]. Thus, a mindfulness-based approach has the potential not only to prevent mental health problems, but also to promote positive well-being and overall health.

### Health Action Process Approach as a Means to Enhance Mindfulness Practice

Given that mindfulness-based training is traditionally delivered face-to-face and requires participants to diligently practice mindfulness daily throughout the training period, adherence to mindfulness practice may be a concern if delivered over the Internet. In this study, in addition to investigating the efficacy of Internet-based mindfulness programs for mental health promotion, we also evaluated the utility of applying principles set out in the Health Action Process Approach (HAPA) in enhancing the effect of mindfulness on mental health outcomes. This was done by comparing its effects on (1) an Internet-based mindfulness program, (2) an Internet-based, HAPA-enhanced mindfulness program, and (3) a waitlist control group. Results were assessed at the end of the 8-week program and at 3-month follow-up.

The HAPA stresses the importance of post-intentional mechanisms in making behavior change. It has been widely applied across many different health behaviors and across a wide range of health conditions to promote behavioral change, including adherence to physical exercise after cardiac rehabilitation [30,31], breast self-examination [32], dietary behaviors [33-36], vaccination adherence [37], and physical activity [35,38].

Given that mind wandering (the opposite of a mindful state) is a habitual tendency in most individuals, it is not uncommon for people new to mindfulness training to become frustrated and confused by the nature of the exercises. Therefore, in the HAPA-enhanced mindfulness condition, we incorporated specific guidelines to enable participants to anticipate difficulties in keeping up the routine and to develop their own corresponding mitigation strategies. Messages on acknowledgment of potential difficulties and encouraging resumption (despite temporary disengagement) were incorporated in the lesson content. Thus, the HAPA-enhanced condition aimed to increase participants’ planning of and effectiveness in their daily exercises, as well as development of coping strategies towards daily obstacles and resumption of practice after setbacks.

Despite a wealth of evidence being established for Internet-based interventions and mindfulness separately, researchers are still at an early stage in empirically evaluating the efficacy of online mindfulness program (eg, [39]), which can potentially be a cost-effective promotion and universal prevention option for adults in the community. This study sought to evaluate such programs in a randomized controlled trial (RCT). We hypothesized that the HAPA-enhanced mindfulness group would demonstrate better outcomes than the basic mindfulness group, and both groups would attain better well-being than waitlist control.

## Methods

### Participants, Design, and Procedure

Participants were recruited from seven local universities in Hong Kong through mass emails, newsletters, and online forums. The general public was welcome to join. All participants were above age 18 and were computer literate. In total, 321 university staff and students signed up for the program. We randomly assigned them into three groups based on a random digit generated by the computer program: HAPA-enhanced mindfulness, basic mindfulness, and waitlist control. The study was approved by the institutional review board of the investigators’ university. See Multimedia Appendix 1 for the CONSORT-EHEALTH checklist [40].

**Figure 1:**
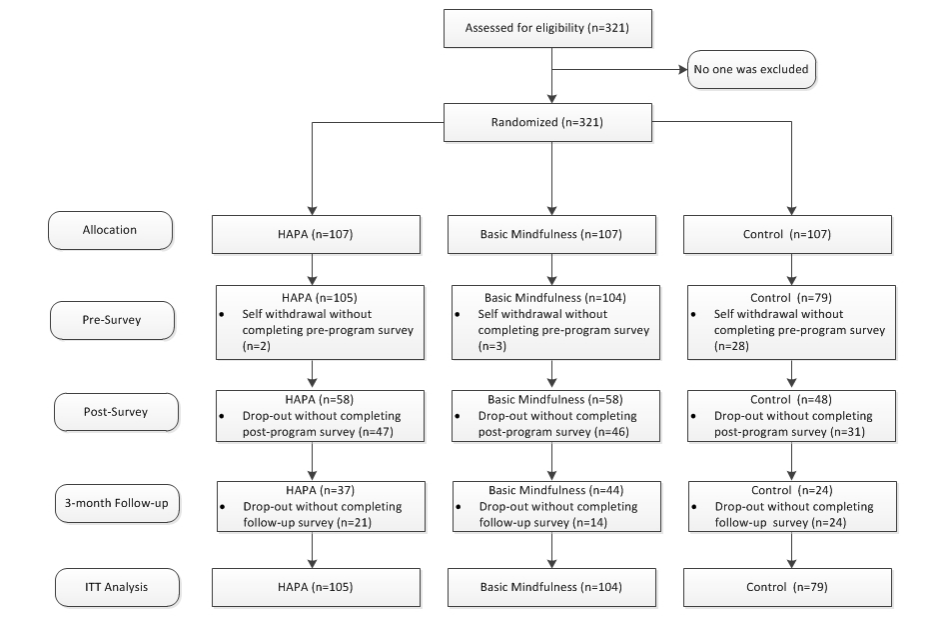
Flowchart of randomization and retention of participants.

While 105 participants from the HAPA-enhanced group and 104 from the basic mindfulness group completed the pre-program survey, only 73.8% (79/107) of participants from the control condition remained to give informed consent. Our sample had a mean age of 22.8 (SD 6.504); the majority (191/286, 66.3%, with 2 missing data) was female. Less than one-fifth (15.6%, 45/285, with 3 missing data) reported having previous experience on mindfulness and related practice. About one-third (34.7%, 100/287, 1 missing data) reported having a religious belief, with most of them being affiliated with Christianity (14%, 40/63), followed by Catholicism and Buddhism, with each accounting for 4% (10/63); 1 person reported to be affiliated with Taoism, and 2 were affiliated with other religions. Chi-square analysis revealed no cross-group differences on previous mindfulness practice and religious affiliation. For age, analysis of variance (ANOVA) indicated that control participants (mean age 21.4, SD 6.04) were significantly younger than the basic mindfulness groups (mean age 24.1, SD 7.61) but not the HAPA-enhanced group (mean age 22.4, SD 5.36; *F*_2,276_=3.99, *P*=.02). As for retention rate at post-program and 3-month follow-up (see Figure 1), the overall retention rates were 51.1% (164/321) and 32.7% (105/321) at post-assessment and 3-month follow-up, respectively.

Participants assigned to the HAPA-enhanced and basic mindfulness groups were invited to attend a 3-hour workshop to provide a basic overview of mindfulness and other logistic details concerning website usage. On a set date, all participants started the online programs, which were similar to the traditional MBSR structure, with the exception of the full-day retreat. The program content and exercises were prepared by the research team based on the MBSR materials used locally by a certified mindfulness trainer with granted permission on usage. The final contents were reviewed by a certified MBSR trainer. New online lesson material was made available weekly, with a total of eight lessons.

**Figure 2:**
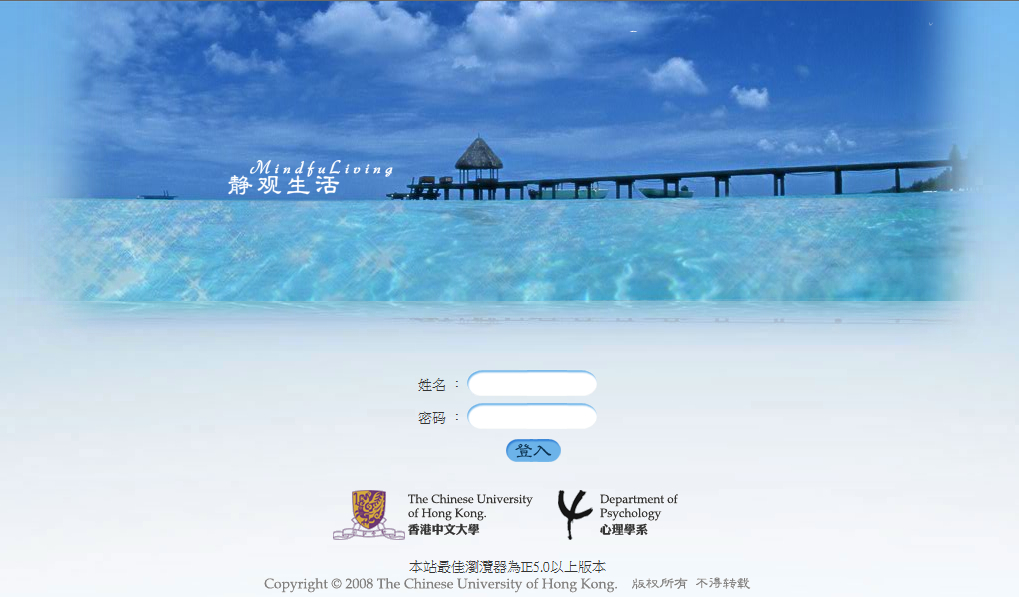
MindfuLiving login page.

**Figure 3:**
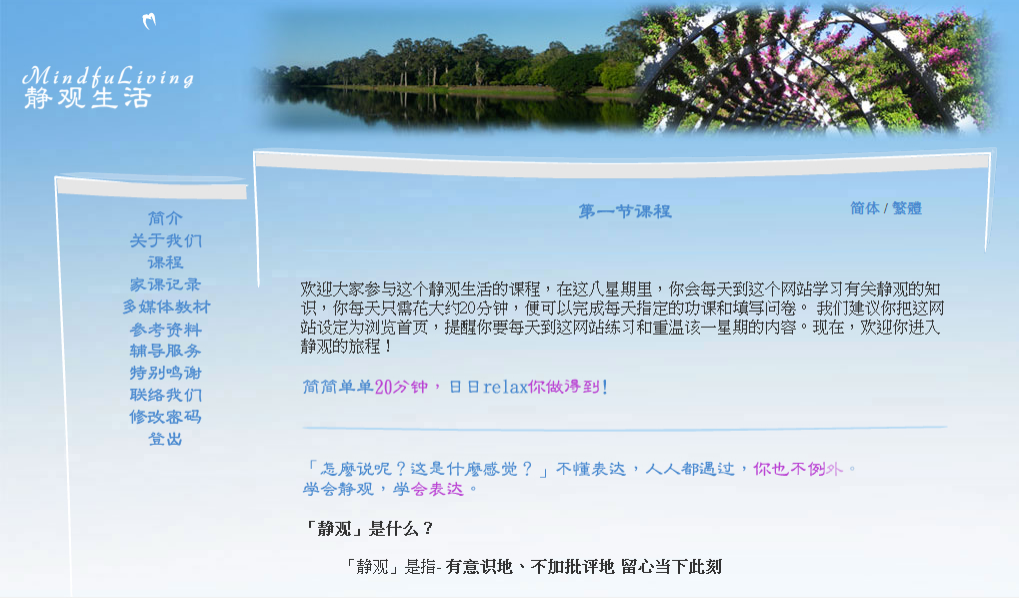
MindfuLiving lesson content.

**Figure 4:**
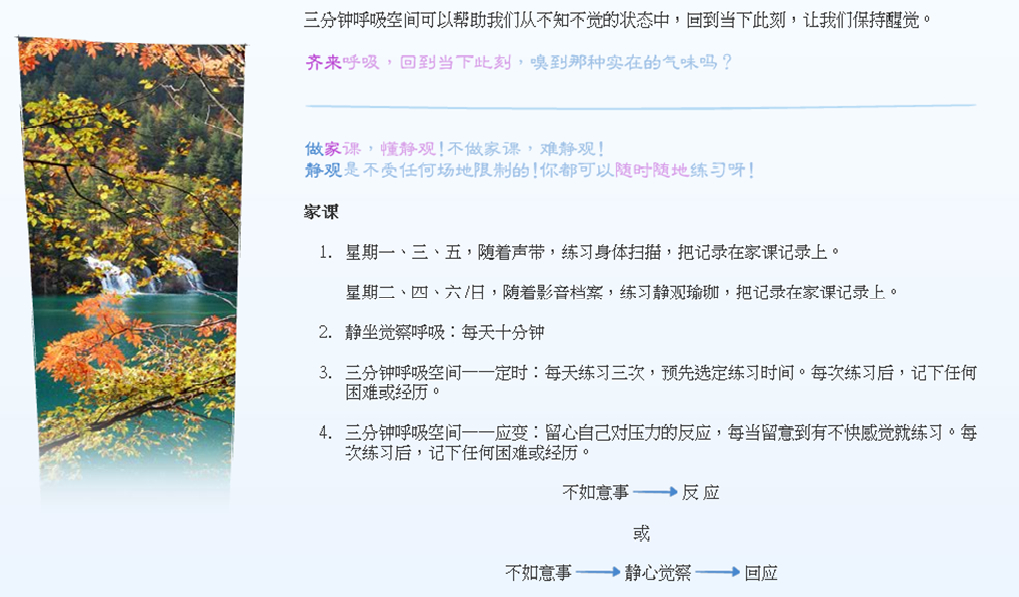
Suggested off-class mindfulness practices.

Participants logged into the assigned website using a unique user identity and password, and their progress was tracked by the system. They were expected to spend an average of 30 minutes per week viewing each lesson, which consisted of an overview of various mindfulness topics, in addition to downloadable meditation audios and videos for mindful stretching exercises, lasting 20-30 minutes each (see Figures 2-4 for screenshots). Participants were asked to conduct formal mindfulness practice for 20-30 minutes daily for 6 days a week. Participants were also encouraged to engage in various mindfulness practices on a daily basis and to report their progress weekly using a log sheet. All materials were presented in Chinese, which was the native language for all participants. Automated emails were sent to participants once a week to remind them about their weekly attendance and regular practice.

All course materials related to teaching mindfulness were identical between HAPA-enhanced and basic mindfulness groups, except that the HAPA-enhanced participants received additional guidance derived from the HAPA model to help them translate intention into action and keep up the exercises despite setbacks. These included:

1. Action and maintenance self-efficacy. Additional self-efficacy statements (eg, “You only have to practice for 20 minutes a day; you can do it!” and “Believe in yourself; keep practicing”) were incorporated in the course materials to motivate participants to turn motivation into action.
2. Action planning. In the weekly homework log sheets, participants had to write down when, where, and how they planned to engage in the assigned mindfulness practice to concretize their practice plan. Statements related to planning (eg, “Mindfulness practice only takes 20 minutes a day; try to plan ahead”) also appeared in the session content to improve their planning.
3. Coping planning. Participants were asked to envisage potential obstacles that might hinder their practice, followed by concrete strategies that they would use to overcome them.
4. Recovery self-efficacy. Reminder statements were added to emphasize the importance of resuming practices when participants lapsed. For example, “If you fell asleep during practice, don’t be discouraged. Just learn to feel your body; you can do it!” Participants were also asked to write down their successful experiences, which served to further reinforce their regular practices.
5. Based on the participants’ responses about their last session’s practice, a variety of pop-up messages appeared in the following session to increase their efficacy. For example, if the participant had practiced only 1-2 days a week, the message was “Keep working hard; you will feel the changes if you practice mindfulness regularly. Using the action and coping planning worksheets and your self-efficacy, you can practice more!” The message for daily practitioner was “Well done, it is great that you are keeping up with your daily mindfulness practice. You have worked hard to beat the odds and difficulties in your practice. Keep believing you can carry on. Well done and continue practicing mindfulness.”

### Measures

#### Mindfulness

The 39-item Five Facets Mindfulness Questionnaire was used to examine participants’ changes in mindfulness level over the course of our program [41]. Participants gave a rating from 1-5 (“never or rarely true” to “very often or always true”) on items covering five mindfulness aspects: non-reactivity, observing, acting with awareness, describing, and non-judging. Sample items include “I perceive my feelings and emotions without having to react to them” (non-reactivity), “When I am walking, I deliberately notice the sensations of my body moving” (observing), “I find it difficult to stay focused on what’s happening in the present” (acting with awareness), “I am good at finding the words to describe my feelings” (describing), and “I criticize myself for having irrational or inappropriate emotions” (non-judging). Cronbach alpha was .88 at baseline, .91 at post-assessment, and .90 at 3-month follow-up.

#### Mental Well-Being

The 5-item World Health Organization Well-Being Index was used to assess participants’ global mental well-being [42]. Sample items included “I have felt cheerful and in good spirits” and “I woke up feeling fresh and rested”. Participants rated on a 6-point Likert scale from 0-5 (“at no time” to “all of the time”). Cronbach alpha was .89 at baseline, .93 at post-assessment, and .93 at follow-up.

#### Life Satisfaction

The Satisfaction With Life Scale [43], together with one item from the Delighted-Terrible (D-T) scale [44] was used to assess participants’ cognitive and affective evaluations of their life. It had a 7-point Likert scale from 1-7 (“strongly disagree” to “strongly agree”) on items such as “In most ways my life is close to my ideal” and “I am satisfied with my life”. Cronbach alpha was .90 at baseline, .91 at post-assessment, and .94 at follow-up.

#### Perceived Stress

To understand the extent to which participants’ personal life situations were perceived as stressful, the 10-item Perceived Stress Scale was used [45]. Participants rated from 0-4 (“never” to “very often”) on items such as “In the last month, how often have you been upset because of something that happened unexpectedly?” and “How often have you felt things were going your way?”. Cronbach’s alpha was .87 at baseline, .80 at post-assessment, and .82 at follow-up.

#### Psychological Symptoms

The short version of the Depression Anxiety Stress Scales (DASS 21) was used to gauge the severity and frequency of symptoms related to depression, anxiety, and stress [46]. It has a 4-point Likert scale ranging from 0-3 (“not applied at all” to “applied very much or most of the time”). Cronbach alpha for each subscale at baseline, post-assessment, and follow-up was as follows: .83, .86, and .87 for the depression subscale, .77, .76, and .85 for the anxiety subscale, and .86, .86, and .88 for the stress subscale.

### Statistical Analyses

Intention-to-treat analysis, with the last observation carried forward, was adopted as a stringent test of efficacy. Repeated measures ANOVA was used to examine outcome changes over time across groups; when the assumption of sphericity was not met, we either adopted the Greenhouse-Geisser adjustment (when epsilon is <.75) or the Huynh-Feldt adjustment (when epsilon ≥.75) to examine the result [47]. Post-hoc analysis was conducted to explore how each group changed over three time points.

## Results

### Effects on Mindfulness

A marginal significant time x group interaction on overall mindfulness was found (*F*_4,568_=2.86, partial eta square=0.02, *P*=.05; the effect was small). While there was no significant main effect of time for control and basic mindfulness groups (*F*_1.67,130.38_=2.66, partial eta square=0.03, *P*=.083; *F*_1.39,142.66_=1.29, partial eta square=0.01, *P*=.27, respectively), the HAPA-enhanced group showed significant improvement from pre- to post-, and such improvement was sustained at 3-month follow-up, with an overall medium effect size, (*F*_1.45,150.72_=7.67, partial eta square=0.07, *P*=.002). No significant interaction effect of individual mindfulness facets was found.

### Effects on Mental Well-Being

Significant time x group interaction was found (*F*_3.76,481.13_=3.13, partial eta square=0.024, *P*=.02). Both basic mindfulness (*F*_1.86,191.14_=10.09, partial eta square=0.09, *P*<.001) and HAPA-enhanced groups (*F*_1.77,184.52_=11.38, partial eta square=0.10, *P*=.001) demonstrated significant improvement from pre- to post- with medium effect size, and improvement was sustained at 3-month follow-up. No significant changes were observed among the waitlist controls across time (*F*_1.39,68.19_=0.254, partial eta square=0.005, *P*=.69).

### Effects on Life Satisfaction

No significant time x group interaction, but a significant main effect of time (*F*_1.61,453.04_=8.58, partial eta square=0.03, *P*=.001) was found. Post-hoc analysis indicated that only the HAPA-enhanced group exhibited a significant main effect of time, with a medium effect size of significant increase in life satisfaction over time, *F*_1.45,150.79_=9.97, partial eta square=0.09, *P<*.001.

### Effects on Perceived Stress and Psychological Symptoms

No significant time x group interaction was found in perceived stress (*F*_3.26,463.89_=1.72, partial eta square=0.01, *P*=.16), depression (*F*_3.52,500.81_=0.60, partial eta square=0.004, *P*=.64), anxiety (*F*_3.56,505.04_=1.71, partial eta square=0.01, *P*=.16), and stress (*F*_3.48,494.74_=1.68, partial eta square=0.012, *P*=.16). Mean scores of depression, anxiety, and stress for all groups across three time points all fell within the normal range of DASS [46]. See Tables 1 and 2 for the comparison of outcomes across three groups.

**Table 1:** Mean (SD) among basic mindfulness, HAPA-enhanced mindfulness, and waitlist control groups on outcomes.

	Basic mindfulness			HAPA-enhanced mindfulness			Control		
	Pre	Post	Follow-up	Pre	Post	Follow-up	Pre	Post	Follow-up
Overall mindfulness	3.13 (0.52)	3.19 (0.53)	3.16 (0.49)	3.08 (0.48)	3.18 (0.49)	3.18 (0.48)	3.18 (0.42)	3.15 (0.43)	3.22 (0.41)
Non-reactivity	3.08 (0.71)	3.01 (0.93)	3.08 (0.73)	3.02 (0.62)	3.07 (0.64)	3.07 (0.63)	3.08 (0.71)	3.02 (0.81)	3.12 (0.69)
Observing	3.11 (0.78)	3.20 (0.91)	3.21 (0.84)	3.20 (0.71)	3.33 (0.76)	3.31 (0.79)	3.21 (0.76)	3.17 (0.82)	3.27 (0.86)
Acting with awareness	3.25 (0.81)	3.30 (0.81)	3.19 (0.76)	3.15 (0.88)	3.22 (0.78)	3.23 (0.79)	3.33 (0.88)	3.29 (0.93)	3.30 (0.88)
Describing	3.17 (0.89)	3.18 (0.79)	3.12 (0.77)	3.10 (0.91)	3.22 (0.84)	3.21 (0.83)	3.25 (0.76)	3.20 (0.73)	3.27 (0.67)
Non-judging	3.00 (0.79)	3.18 (0.88)	3.19 (0.81)	2.91 (0.73)	3.05 (0.75)	3.09 (0.81)	3.03 (0.75)	3.04 (0.75)	3.08 (0.75)
Mental well-being	13.22 (5.07)	15.17 (5.31)	14.47 (5.21)	12.76 (5.65)	14.25 (5.50)	14.51 (5.05)	16.58 (5.29)	16.56 (5.36)	16.22 (5.74)
Life satisfaction	4.16 (1.34)	4.46 (1.32)	4.37 (1.40)	3.96 (1.40)	4.24 (1.36)	4.33 (1.38)	4.55 (1.16)	4.57 (1.30)	4.64 (1.32)
Perceived stress	1.72 (0.60)	1.65 (0.53)	1.70 (0.53)	1.80 (0.63)	1.70 (0.61)	1.67 (0.62)	1.63 (0.70)	1.62 (0.70)	1.63 (0.70)
Depression	6.10 (6.33)	5.69 (6.75)	6.81 (7.16)	6.95 (7.67)	6.51 (7.37)	6.88 (7.70)	5.47 (7.55)	5.85 (7.74)	5.90 (8.12)
Anxiety	6.70 (5.21)	6.56 (5.40)	7.29 (6.36)	7.49 (6.63)	6.27 (6.63)	6.46 (6.60)	6.20 (8.02)	5.67 (7.26)	5.72 (7.64)
Stress	11.43 (8.51)	10.29 (8.74)	11.50 (8.93)	12.44 (9.21)	10.55 (9.05)	10.72 (9.01)	9.52 (9.45)	9.44 (9.23)	9.19 (9.35)

**Table 2:** Effect size (Cohen’s *d*) among basic mindfulness, HAPA-enhanced mindfulness, and waitlist control groups on outcomes.

	Basic mindfulness		HAPA-enhanced mindfulness		Control	
	Pre & post	Pre & follow-up	Pre & post	Pre & follow-up	Pre & post	Pre & follow-up
Overall mindfulness	0.08	0.04	0.25	0.19	-0.07	0.10
Non-reactivity	-0.11	-0.06	0.08	0.08	-0.08	0.06
Observing	0.07	0.13	0.24	0.17	-0.03	0.10
Acting with awareness	0.02	-0.13	0.05	0.05	-0.04	0.03
Describing	-0.01	-0.06	0.01	0.11	-0.07	0.03
Non-judging	0.21	0.23	0.27	0.27	0.01	0.07
Mental well-being	0.35	0.20	0.24	0.24	-0.21	-0.22
Life satisfaction	0.18	0.16	0.22	0.26	0.02	0.07
Perceived stress	-0.09	-0.04	-0.20	-0.16	-0.01	0.00
Depression	-0.04	0.10	-0.09	0.02	0.05	0.05
Anxiety	0.00	0.13	-0.20	-0.10	-0.05	-0.05
Stress	-0.64	-0.51	-0.77	-0.68	-0.45	-0.44

### Correlation Between the Enhancement of Mindfulness and Mental Well-Being Improvement

After the 8-week program, for both basic mindfulness and HAPA-enhanced groups, significant correlations between the change scores of mental well-being and the change scores of mindfulness were found (*r*=.55, *P*<.001 and *r*=.58, *P*<.001, respectively). However, no significant correlation was found for waitlist controls (*r*=.21, *P*=.14), and the difference in correlations was significant between waitlist controls with basic mindfulness (*z* statistics=2.29*, P*=.02) and HAPA-enhanced groups (*z* statistics=2.55*, P*<.001) [48]. At follow-up, such correlation remained significant for HAPA-enhanced (*r*=.59, *P*<.001) and basic mindfulness groups (*r*=.43, *P*<.001) and became marginally significant for waitlist controls (*r*=.28, *P*=.051). However, the difference in correlations at follow-up between waitlist controls was not significant with basic mindfulness (z statistics=0.99*, P*=.32) but remained significant with HAPA-enhanced groups (*z* statistics=2.25*, P*=.02).

### Mindfulness Practice Between Groups

In terms of time commitment on practices, based on the number of days people spent on their home practice during the 8-week program, no significant difference was observed between the basic mindfulness (mean 4.34, SD 3.78) and HAPA-enhanced groups (mean 4.18, SD 2.55); *t*_81_=0.24, *P*=.81.

## Discussion

### Principal Results

This study demonstrated the effectiveness of Internet-based mindfulness intervention in promoting mindfulness and mental well-being of adults in the community, particularly when the program was primed with efficacy and planning components from the Health Action Process Approach. Consistent with previous research that demonstrated the benefits of face-to-face MBSR programs for the general populations [49,50], and recent studies on preliminary evidence and feasibility of brief, online mindfulness courses [39,51], this study further demonstrated that mindfulness can be cultivated over the Internet and such online training can promote mental well-being in an RCT. With the Internet being highly accessible nowadays, Internet-based mindfulness interventions can be a convenient and effective means of promoting public mental health.

Unlike the traditional face-to-face mindfulness-based training programs (eg, MBSR) that pose a heavy time demand on the participants (ie, eight weekly 2.5 hour sessions, 45 minutes daily of formal mindfulness practice for 6 days a week, and a full-day retreat), this program required participants to complete eight 30-minute online sessions, in addition to having 30 minutes of formal mindfulness practice daily for 6 days a week. Previous meta-analysis has found no significant correlation between in-class contact hours and effect sizes of psychological outcomes for both non-clinical and clinical populations and has suggested the possibility of adapting the class time for future practice [52]. Our findings supported the reduction of time commitment for mindfulness practice in providing promising effects on mental well-being of adults in the wider community. In light of the fact that the general public may be less motivated and committed to devoting time for their mental health, Internet-based mindfulness with reduced time demands may be a viable substitute. Moreover, the accessibility, convenience, affordability, and anonymity of Internet-based mindfulness intervention may be regarded as added values that may be appealing to the public, compared with face-to-face mindfulness programs.

This study’s findings also demonstrated that an Internet-based mindfulness program enhanced with HAPA elements could better help participants enhance their mindfulness and mental well-being than its basic form. A slightly stronger relationship between mindfulness with mental well-being was also observed in the HAPA-enhanced group. Results suggested that the supplementary messages intended to increase participants’ planning and practice efficacy and the strategies to help them deal with obstacles may enhance the effects of the program for them. Although the HAPA-enhanced group did not spend more time on practicing than the basic mindfulness group, the former seemed to be able to get more out of their practice. Future research should further examine the mediating roles of HAPA components directly (eg, action self-efficacy, recovery self-efficacy, action, and coping planning) in order to tailor the delivery of online mindfulness training to maximize gains for the general public.

### Limitations

Because the sample focused on university students and staff, the findings may not be generalizable to the general population. Future studies should extend this program to the general public to investigate its efficacy to a wider population. Second, close to half of the participants were lost at post-program assessment and two-thirds lost at 3-month follow-up. Although high attrition rate has been a perennial problem for Internet-based interventions [53-55], with an average of 74.7% attrition observed, the remaining participants may represent a select and motivated group such that their results may not be generalizable to the general public. Furthermore, research has found that those who participated longer in the program and completed more sessions online were found to have better outcomes [56]. Future studies should obtain feedback from participants, especially from those who have dropped out to elucidate their reasons for dropping out versus completing the program. This information can assist researchers in exploring ways to enhance continued participation in Internet-based interventions. Possible strategies may include the inclusion of online support groups or chat rooms among participants to enhance a sense of membership and interpersonal support or the use of technicians in providing telephone support to minimize attrition [16,57,58]. Finally, we used waitlist control as a comparison group. Given that cognitive-behavioral Internet-based interventions have shown to be effective for various health conditions and populations [59,60], future research could consider comparing online cognitive-behavioral and mindfulness training programs to further investigate their differential effects, mechanisms of change, and suitability for different kinds of participants [61].

### Conclusions

Notwithstanding these limitations, this study demonstrated the usefulness of health behavioral theory in enhancing changes in mindfulness and mental well-being among adults in an Internet-based program, and the application of Internet-based mindfulness training in promoting public mental health.

## Multimedia Appendix 1

CONSORT-EHEALTH checklist V1.6.2 [61].

## Abbreviations

ANOVA: analysis of variance
DALY: disability-adjusted life years
DASS: Depression Anxiety Stress Scales
HAPA: Health Action Process Approach
MBCT: mindful-based cognitive therapy
MBSR: mindfulness-based stress reduction
RCT: randomized controlled trial
