# Supplementary material for: Enhancing Web-Based Mindfulness Training for Mental Health Promotion With the Health Action Process Approach: Randomized Controlled Trial
Source: J Med Internet Res. 2015 Jan 19;17(1):e8. doi: 10.2196/jmir.3746 (PMC4319090; doi:10.2196/jmir.3746)
Supplement: Supplementary file 1 [file jmir_v17i1e8_app1.pdf]

CONSORT-EHEALTH Checklist V1.6.2 Report

(based on CONSORT-EHEALTH V1.6), available at [http://tinyurl.com/consort-ehealth-v1-6].

3746

Date completed

4/29/2014 3:30:28

by

Winnie W. S. Mak

Randomized controlled trial of web-based mindfulness training for mental health promotion in the general public: Enhancements with the Health Action Process Approach

TITLE

1a-i) Identify the mode of delivery in the title

Yes. "Randomized controlled trial of web-based mindfulness training for mental health promotion in the general public: Enhancements with the Health Action Process Approach"

1a-ii) Non-web-based components or important co-interventions in title

No. Our study does not involve non-web-based component.

1a-iii) Primary condition or target group in the title

No. Our study targets general public rather than a specific group of the population.

ABSTRACT

1b-i) Key features/functionalities/components of the intervention and comparator in the METHODS section of the ABSTRACT

Yes. "This study evaluated the efficacy of two internet-based interventions (basic mindfulness and Health Action Process Approach (HAPA)-enhanced mindfulness) with waitlist control. HAPA principles were used to enhance participants' efficacy and planning."

1b-ii) Level of human involvement in the METHODS section of the ABSTRACT

Yes. "The basic and HAPA-enhanced groups completed the 8-week fully automated mindfulness training online."

1b-iii) Open vs. closed, web-based (self-assessment) vs. face-to-face assessments in the METHODS section of the ABSTRACT

Yes. "Participants were recruited online and offline among local universities."

1b-iv) RESULTS section in abstract must contain use data

Yes. "Three hundred and twenty-seven university students and staff were randomly assigned to three conditions. The basic (n=119) and HAPA-enhanced (n=134) groups completed the 8-week fully automated mindfulness training online. All participants (including control, n=107) were asked to complete a questionnaire pre-program, post-program, and at 3-month follow-up."

1b-v) CONCLUSIONS/DISCUSSION in abstract for negative trials

Yes. "The HAPA-enhanced group showed significantly higher levels of mindfulness from pre- to post-, and such improvement was sustained at follow-up. Both the basic and HAPA-enhanced mindfulness groups showed better mental well-being from pre- to post-, and improvement was sustained at 3-month follow-up."

INTRODUCTION

2a-i) Problem and the type of system/solution

Yes. The objective of this study is to explore the efficacy of teaching mindfulness over the internet without the traditional face-to-face setting. In addition, "we also evaluated the utility of applying principles set out in the Health Action Process Approach (HAPA) in enhancing the effect of mindfulness on mental health outcomes."

2a-ii) Scientific background, rationale: What is known about the (type of) system

Yes. "Internet-based interventions have been shown to be effective in the prevention of depression and anxiety for both research trial participants and public registrants (Andersson et al., 2005; Christensen & Griffiths, 2002; Christensen, Griffiths, & Jorm, 2004; Christensen, Griffiths, Korten, Brittliffe, & Groves, 2004; Clarke et al., 2005; Spek et al., 2007; Titov et al., 2009). The effectiveness of internet-based mental health promotion was also demonstrated in community samples in Western countries (Andersson et al., 2005; Christensen, Griffiths, & Jorm, 2004; Christensen, Griffiths, Korten, et al., 2004; Clarke et al., 2005; Titov et al., 2009); however, no study to date has evaluated internet-based mental health promotion programs in Asia."

METHODS

3a) CONSORT: Description of trial design (such as parallel, factorial) including allocation ratio

Yes. This study aims to evaluate the efficacy of the web-based HAPA-enhanced mindfulness, basic mindfulness programs against waitlist control. "We hypothesize HAPA-enhanced mindfulness group to demonstrate better result than basic mindfulness group, and both groups should attain better well-being than waitlist control."

3b) CONSORT: Important changes to methods after trial commencement (such as eligibility criteria), with reasons

No. No important change to methods after trial commencement.

3b-i) Bug fixes, Downtimes, Content Changes

No system change after trial commenced.

4a) CONSORT: Eligibility criteria for participants

Yes. "Eligibility criteria include at least 18 years of age, are computer and Internet literate, and have easy access to the Internet."

4a-i) Computer / Internet literacy

Yes. "All participants were computer and Internet literate."

4a-ii) Open vs. closed, web-based vs. face-to-face assessments:

Yes. "Participants were recruited from seven local universities in Hong Kong through mass emails, newsletters, and online forums." "Participants assigned to the HAPA-enhanced and basic mindfulness groups were invited to attend a 3-hour workshop to provide a basic overview of mindfulness and other logistic details concerning website usage. At the end of the workshop, each of the participants was given a unique user identification and password to access the website. They are allowed to change the password upon their first log-in."

4a-iii) Information giving during recruitment

Yes. "When participants signed up for the participation in the study, they were informed that it is a research study where they will be randomly assigned to one of the three conditions: HAPA-enhanced mindfulness, basic mindfulness, and waitlist control. We randomly assigned them into three groups based on a random digit generated by the computer program. All participants were informed that they need to complete three sets of questionnaire at pre-program, post-program, and at three-month follow-up. For control participants, it would be at baseline, 8 weeks post-baseline, and 20 weeks post-baseline. Participants assigned to the former two conditions were asked to log into the website for six days a week for eight weeks to review the information, do the exercises, and report their well-being. Those in the control condition were given access to the website five months later. The study was approved by the institutional review board of the investigators' university."

4b) CONSORT: Settings and locations where the data were collected

Yes. All data were collected online.

4b-i) Report if outcomes were (self-)assessed through online questionnaires
Yes. Participants filled out the all questionnaires online via Qualtrics system.
4b-ii) Report how institutional affiliations are displayed
Yes. During informed consent, participants were informed about that the study was conducted by the Department of Psychology, at the Chinese University of Hong Kong. At the bottom of the sign-in page, the logo and name of the university was displayed.
5) CONSORT: Describe the interventions for each group with sufficient details to allow replication, including how and when they were actually administered
5-i) Mention names, credential, affiliations of the developers, sponsors, and owners
Yes. The course material was developed by the authors. Website was developed by Frasertec Limited.
5-ii) Describe the history/development process
Yes. A focus group was conducted to understand users' opinion on online learning and their perspective on mindfulness. Their feedback was eventually incorporated into the courseware development.
5-iii) Revisions and updating
No. No major change was performed after website launched.
5-iv) Quality assurance methods
Yes. Functional and user-acceptance tests on course content and surveys were conducted prior to website release.
5-v) Ensure replicability by publishing the source code, and/or providing screenshots/screen-capture video, and/or providing flowcharts of the algorithms used
Yes. Sreenshots are attached.
5-vi) Digital preservation
No. Since no funding can be set aside to keep the website up and running, www.mindfuliving.hkit is no longer accessible. Only screenshot is available.
5-vii) Access
Yes. Participants accessed the application with an URL, "...each of the participants was given a unique user identification and password to access the website". The website is no longer accessible to the public after study completion.
5-viii) Mode of delivery, features/functionalities/components of the intervention and comparator, and the theoretical framework
Yes, "Mindfulness is an approach that focuses on the cultivation of consciousness, non-judgmental awareness in the unfolding of events in the present moment (Kabat-Zinn, 1990). " "During the 8-week program, mindfulness is cultivated through exercises such as sitting meditation, body scan, and stretching exercises (Kabat-Zinn, 1990). In addition to formal practices, participants are encouraged to practice mindfulness during daily activities (e.g., walking or eating)." For the HAPA-enhanced group, in order to enhance the post-intentional change in accordance to the HAPA principles, feedback was provided based on user's input. For example, if a user reported below-than-average practice frequencies, s/he would be asked to think what their obstacles were and ways to overcome them. And for those who reported high adherence to our recommended practice dosage, they would see a statement of encouragement. Participants were expected to spend 30 minutes on each lesson, and the course material was developed based on this time span.
5-ix) Describe use parameters
Yes. Participants "were expected to spend an average of 30 minutes per week viewing each lesson, which consisted of an overview of various mindfulness topics, in addition to downloadable meditation audios and videos for mindful stretching exercises, lasting 20–30 minutes each. Participants were asked to conduct formal mindfulness practice for 20–30 minutes daily for six days a week. Participants were also encouraged to engage in various mindfulness practices on a daily basis and to report their progress weekly using a log sheet". Adherence to practice dosage was ad libitum.
5-x) Clarify the level of human involvement
Yes. "Participants assigned to the HAPA-enhanced and basic mindfulness groups were invited to attend a 3-hour workshop to provide a basic overview of mindfulness and other logistic details concerning website usage." That was the only human involvement in our study.
5-xi) Report any prompts/reminders used
Yes. Participants "received email reminder once a week about the release of the upcoming lesson."
5-xii) Describe any co-interventions (incl. training/support)
No. There's no co-intervention.
6a) CONSORT: Completely defined pre-specified primary and secondary outcome measures, including how and when they were assessed
Yes. Only primary outcome measures were applicable. "All participants (including control, n=107) were asked to complete an online questionnaire pre-program, post-program, and at 3-month follow-up." Primary outcome measures included: mindfulness, mental well-being, life satisfaction, perceived stress, and psychological symptoms.
6a-i) Online questionnaires: describe if they were validated for online use and apply CHERRIES items to describe how the questionnaires were designed/deployed
Yes. "Functional and user-acceptance tests on course content and surveys were conducted prior to website release."
6a-ii) Describe whether and how "use" (including intensity of use/dosage) was defined/measured/monitored
Yes. "User login and logout time were captured by the backend system. Practice frequency and duration were self-reported by participants."
6a-iii) Describe whether, how, and when qualitative feedback from participants was obtained
No. We didn't collect qualitative data intentionally. However, participants could email the research team on feedback and queries.
6b) CONSORT: Any changes to trial outcomes after the trial commenced, with reasons
No. There was no change to trial outcomes after the trial commenced.
7a) CONSORT: How sample size was determined
7a-i) Describe whether and how expected attrition was taken into account when calculating the sample size
Yes. Based on previous studies in internet interventions, we expected a 70% attrition at follow-up. A sample size of 114 was needed to achieve enough statistical power (0.95) to reject null the hypothesis, using repeated measures ANOVA. 380 people were needed to recruited upfront to account for attrition. During implementation, "three hundred and sixty university staff and students signed up for the program. After informed consent, 327 participants remained."
7b) CONSORT: When applicable, explanation of any interim analyses and stopping guidelines
No. There was no interim analysis
8a) CONSORT: Method used to generate the random allocation sequence
Yes. Participants were randomly assigned "into three groups based on a random digit generated by the computer program".
8b) CONSORT: Type of randomisation; details of any restriction (such as blocking and block size)
Yes. "We randomly assigned them into three groups based on a random digit generated by the computer program (i.e. true randomization)."
9) CONSORT: Mechanism used to implement the random allocation sequence (such as sequentially numbered containers), describing any steps taken to conceal the sequence until interventions were assigned

Yes. Randomization was performed automatically by the website programming logic after participants gave consent to participating in the study. “No one had prior knowledge on condition assignment till randomization was performed.” No one had prior knowledge regarding which condition a participant got assigned to till the assignment was completed.

10) CONSORT: Who generated the random allocation sequence, who enrolled participants, and who assigned participants to interventions

Yes. Random allocation was performed programmatically by the web program implementation. "We randomly assigned them into three groups based on a random digit generated by the computer program (i.e. true randomization)."

11a) CONSORT: Blinding - If done, who was blinded after assignment to interventions (for example, participants, care providers, those assessing outcomes) and how

11a-i) Specify who was blinded, and who wasn’t

Yes. "Participants got assigned to intervention groups (i.e. HAPA-enhanced, basic) had no knowledge which specific intervention group they were in. Participants in wait-list control were informed about their status. Researchers were blinded to group assignments."

11a-ii) Discuss e.g., whether participants knew which intervention was the “intervention of interest” and which one was the “comparator”

Yes. Due to the subtle difference between HAPA-enhanced and basic mindfulness conditions, participants would be very unlikely to find out which specific intervention they were in. Waitlist participants, on the other hand, would know they were not receiving the intervention."Participants got assigned to intervention groups (i.e. HAPA-enhanced, basic) had no knowledge which specific intervention group they were in. Participants in wait-list control were informed about their status."

11b) CONSORT: If relevant, description of the similarity of interventions

Yes.

“All course materials related to teaching mindfulness were identical between HAPA-enhanced and basic mindfulness groups, except that the HAPA-enhanced participants would receive additional guidance derived from the HAPA model to help them translate intention into action and keep up the exercises despite setbacks. These included:
1) Action and maintenance self-efficacy. Additional self-efficacy statements (e.g. “You only have to practice for 20 minutes a day; you can do it!” and “Believe in yourself; keep practicing”) were incorporated in the course materials to motivate participants to turn motivation into action.
2) Action planning. In the weekly homework log sheets, participants had to write down when, where, and how they planned to engage in the assigned mindfulness practice to concretize their practice plan. Statements related to planning (e.g. “Mindfulness practice only takes 20 minutes a day; try to plan ahead”) also appeared in the session content to improve their planning.
3) Coping planning. Participants were asked to envisage potential obstacles that might hinder their practice, followed by concrete strategies that they would use to overcome them.
4) Recovery self-efficacy. Reminder statements were added to emphasize the importance of resuming practices when participants lapsed. For example, “If you fell asleep during practice, don’t be discouraged. Just learn to feel your body; you can do it!” Participants were also asked to write down their successful experiences that served to further reinforce their regular practices.
5) Based on the participants’ responses about their last session’s practice, a variety of pop-up messages appeared in the following session to increase their efficacy. For example, if the participant had only practiced 1–2 days a week, the message was “Keep working hard; you will feel the changes if you practice mindfulness regularly. Using the action and coping planning worksheets and your self-efficacy, you can practice more!” The message for daily practitioner was: “Well done, it is great that you are keeping up with your daily mindfulness practice. You have worked hard to beat the odds and difficulties in your practice. Keep believing you can carry on. Well done and continue practicing mindfulness.””

12a) CONSORT: Statistical methods used to compare groups for primary and secondary outcomes

Yes. “Repeated measures ANOVA was used to examine outcome changes over time across groups. Post-hoc analysis was conducted to explore how each group changed over three time-points.”

12a-i) Imputation techniques to deal with attrition / missing values

Yes. “Intention-to-treat (ITT) analysis, with the last observation carried forward, was adopted as a stringent test of efficacy.”

12b) CONSORT: Methods for additional analyses, such as subgroup analyses and adjusted analyses

Yes. Subgroup analysis was only performed when time x group interaction effect was found during repeated measures ANOVA.

RESULTS

13a) CONSORT: For each group, the numbers of participants who were randomly assigned, received intended treatment, and were analysed for the primary outcome

Yes.

The number of participants in each condition is as follow:
-Waitlist control: 107
-Basic mindfulness: 119
-HAPA-enhanced : 134

13b) CONSORT: For each group, losses and exclusions after randomisation, together with reasons

Yes. This will be reflected by the CONSORT flow diagram.

13b-i) Attrition diagram

Yes. An attrition diagram is available on the manuscript.

14a) CONSORT: Dates defining the periods of recruitment and follow-up

Yes. Participants were recruited during September 2008 to January 2009. 3-month follow-up assessments were conducted during March 2009 to June 2009.

The study was conducted from September 2008 to March 2009. Two waves of identical online program, named “MindfuLiving”, were implemented in September 2008 and February 2009. Emails about the program were disseminated to various mailing lists for each of the seven local universities, forums and newsgroups for university students and staff, all of whom were computer literate and had easy computer access. A total of 326 participants joined the program. Upon informed consent, participants were randomly assigned to waitlist control and treatment group. We assigned one-fourth of the participants to the control and the rest to the treatment group. A total of 123 participants were assigned to the treatment group in September 2008 and 124 participants were assigned to the treatment group in February 2009.

14a-i) Indicate if critical “secular events” fell into the study period

No. There was no secular events fell into the study period.

14b) CONSORT: Why the trial ended or was stopped (early)

No. The trial did not end early.

15) CONSORT: A table showing baseline demographic and clinical characteristics for each group

No.

The content of the mindfulness intervention was developed based on Jon Kabat Zinn's mindfulness program (Kabat-Zinn, 1990). Therefore, the concept of care provider is not applicable in this study.

15-i) Report demographics associated with digital divide issues

Yes.

All participants (N=327) have graduated from high school. 88.7% were college students. The rest was working individuals. Among the non-college sample, 78.2% did graduate from college. "Our sample had a mean age of 25 (SD = 9.41), the majority (65.2%) were female." No income or socioeconomic status information was collected.

16a) CONSORT: For each group, number of participants (denominator) included in each analysis and whether the analysis was by original assigned groups

16-i) Report multiple "denominators" and provide definitions

Yes. 360 people signed up, 327 agreed to participate by giving their informed consent and filled out the pre-program survey. 175 completed the 8-week program, and 113 agreed to complete the 3-month follow-up survey.

16-ii) Primary analysis should be intent-to-treat

Yes. "Intention-to-treat (ITT) analysis, with the last observation carried forward", was adopted for primary analysis.

17a) CONSORT: For each primary and secondary outcome, results for each group, and the estimated effect size and its precision (such as 95% confidence interval)

Yes.

For effects on mindfulness, "a significant time x group interaction on overall mindfulness was found [$F(4, 646) = 3.097$, partial $\eta2 = .019$, $p=.015$, the effect was small]. While there was no significant main effect of time for control and basic mindfulness groups ([$F(2,156)=2.662$, partial $\eta2 = .033$, $p=.073$] , [$F(2,230)=1.355$, partial $\eta2 = .012$, $p=.26$], respectively), the HAPA-enhanced group showed significant improvement from pre- to post-, and such improvement was sustained at 3-month follow-up, with an overall medium effect size,[$F(2,262)=10.91$, partial $\eta2 =.096$, $p=<.001$]. No significant interaction effect of individual mindfulness facets was found."

For effect on mental well-being, "significant time x group interaction was found [$F(4,588)=2.893$, partial $\eta2=.019$, $p=.022$]. Both basic mindfulness [$F(2,114)=9.80$, partial $\eta2=.147$, $p<.001$] and HAPA-enhanced groups [$F(2,130)=11.46$, partial $\eta2=.15$, $p<.001$] demonstrated significant improvement from pre- to post- with large effect size, and improvement was sustained at 3-month follow-up. No significant changes were observed among the waitlist controls across time [$F(2,48)=.151$, partial $\eta2 =.006$, $p=.86$]."

For effects on life satisfaction, "no significant time x group interaction, but a significant main effect of time [$F(2,320)=6.819$, partial $\eta2=.041$, $p=.001$] was found. Post-hoc analysis indicated that only the HAPA-enhanced group exhibited significant main effect of time, with a large effect size, $F(2,130)=8.811$, partial $\eta2=.119$, $p<.001$."

For effect on perceived stress and psychological symptoms, "no significant time x group interaction was found in perceived stress [$F(4,646)=1.185$, partial $\eta2=.007$, $p=.316$], depression [$F(4,644)=.742$, partial $\eta2=.005$, $p=.563$], anxiety [$F(4,644)=1.489$, partial $\eta2=.009$, $p=.204$], and stress [$F(4,644)=1.757$, partial $\eta2=.011$, $p=.136$]."

17a-i) Presentation of process outcomes such as metrics of use and intensity of use

Yes. "No significant difference was observed between the basic mindfulness and HAPA-enhanced groups on their time and intensity of home practice during the 8-week programs." Basic mindfulness group spent an average of 3.9 hours/week on after-class mindfulness practices, versus 3.7 hours/week for HAPA-enhanced group. There was no significant difference between the two groups, $t(178)=0.40$, $p=0.69$.

17b) CONSORT: For binary outcomes, presentation of both absolute and relative effect sizes is recommended

No. Binary outcome is not applicable to our study which assessed mental well-being.

18) CONSORT: Results of any other analyses performed, including subgroup analyses and adjusted analyses, distinguishing pre-specified from exploratory

No. Subgroup analysis was not applicable to this study.

18-i) Subgroup analysis of comparing only users

No. Not applicable.

19) CONSORT: All important harms or unintended effects in each group

No.

No harmful effect was exerted on participants.

19-i) Include privacy breaches, technical problems

No.

No privacy breach, technical problems, or other unexpected incidents happened throughout the study.

19-ii) Include qualitative feedback from participants or observations from staff/researchers

No qualitative feedback concerning unintended/unexpected effects was gathered in this studyreported by participants or staffs/researchers

DISCUSSION

20) CONSORT: Trial limitations, addressing sources of potential bias, imprecision, multiplicity of analyses

20-i) Typical limitations in ehealth trials

Yes. "A few limitations should be noted for the present study. Close to half of the participants were lost at post-program assessment and two-thirds lost at three-month follow-up. Although high attrition rate had been a perennial problem for internet-based interventions (Christensen, Griffiths, & Korten, 2002; Farvolden, Denisoff, Selby, Bagby, & Rudy, 2005; Tumur, Kaltenthaler, Ferriter, Beverley, & Parry, 2007), with an average of 74.7% attrition observed, the remaining participants may represent a selected and motivated group that their results may not be generalizable to the general public. Furthermore, research has found that those who participated longer in the program and completed more sessions online were found to have better outcomes (Christensen, Griffiths, Groves, & Korten, 2006). Future research on internet-based interventions should explore means to enhance their continued participation. Possible strategies may include the inclusion of online support groups or chat rooms among participants to enhance a sense of membership and interpersonal support or the use of technicians in providing telephone support to minimize attrition (Robinson et al., 2010; Titov, Andrews, Choi, Schwencke, & Johnston, 2009; Titov, Andrews, Schwencke, Solley, Johnston, & Robinson, 2009).

In addition, the present study used waitlist control as a comparison group. Given that cognitive-behavioral, internet-based interventions have shown to be effective for various health conditions and populations (Christensen, Griffiths, Mackinnon, & Brittliffe, 2006; Cuijpers, van Straten, & Andersson, 2008), future research could consider comparing online cognitive-behavioral and mindfulness training programs to further investigate on their differential effects, mechanisms of change, and suitability for what kind of participants (Danaher & Seeley, 2009)."

21) CONSORT: Generalisability (external validity, applicability) of the trial findings

21-i) Generalizability to other populations

Yes. "The present study demonstrated the usefulness of health behavioral theory in enhancing changes in mindfulness and mental well-being among adults in an internet-based program, and the application of internet-based mindfulness training in promoting public mental health."

21-ii) Discuss if there were elements in the RCT that would be different in a routine application setting

Yes. The routine application setting will be the identical to this study's. The only exception is there will be no waitlist control group.

22) CONSORT: Interpretation consistent with results, balancing benefits and harms, and considering other relevant evidence

22-i) Restate study questions and summarize the answers suggested by the data, starting with primary outcomes and process outcomes (use)

Yes. “The present study demonstrated the effectiveness of internet-based mindfulness intervention in promoting mindfulness and mental well-being of adults in the community, particularly when the program was primed with efficacy and planning components from the Health Action Process Approach.”

22-ii) Highlight unanswered new questions, suggest future research

Yes. “Future research should further examine the mediating roles of HAPA components directly (e.g., action self-efficacy, recovery self-efficacy, action and coping planning) in order to tailor the delivery of online mindfulness training to maximize gains for the general public.”

Other information

23) CONSORT: Registration number and name of trial registry

Yes. Registration Number: ChiCTR-TRC-12002954

24) CONSORT: Where the full trial protocol can be accessed, if available

Yes.
www.chictr.org

25) CONSORT: Sources of funding and other support (such as supply of drugs), role of funders

Yes.
This study was funded by the Direct Grant of the Chinese University of Hong Kong (Ref. No. 2020978)

X26-i) Comment on ethics committee approval

Yes. “The study was approved by the institutional review board of the investigators’ university.”

x26-ii) Outline informed consent procedures

Yes. Participants clicked “I agree” button on the consent page to indicate their understanding and agreement in participating in our study.

X26-iii) Safety and security procedures

Yes. Registration and login data transmitted online was encrypted using HTTPS protocol. All data were kept confidential and only accessible by the research investigators of this study. Participants could contact the research team via email.

X27-i) State the relation of the study team towards the system being evaluated

Yes.The authors are also the developers of the intervention.
